# Observations on Mr. Earle's Fracture-Bed, &c.

**Published:** 1824-11

**Authors:** E. Harrold

**Affiliations:** Surgeon.


ART. V.—*Observations on* Mr. EARLE's *Fracture-Bed, &c.* By E. HARROLD, Esq. Surgeon.

AT page 12, No. 17 of the Old, or No. 1 of the New Series of the Medico-Chirurgical Review, in the analysis of Dr. OLLIVIER's work on *Diseases of the Spinal Marrow*, the Reviewers have referred to my case of Fractured Vertebræ; and, as there is some error in their statement, which may affect the view of these important and interesting cases, allow me just to set the matter right.

My patient did not die from a disease of the *sacrum*, over which the slough had been situated ; but from a disease of the *ischium*, produced by his bruising the integuments covering the tuberosity of that bone, in daily letting himself, unassisted, down stairs, long after the union of the fractured vertebræ, and the perfect and permanent cicatrization of the wound over the sacrum. (See *Medical and Physical Journal*, vol. 26, p. 371.)

Your account of Mr. EARLE's "Practical Observations in Surgery," and the accompanying wood-cut of his Fracture-bed, contained in the Medico-Chirurgical Review, excited my curiosity to see the original work; which, I am free to confess, surprised me exceedingly.

I shall be as brief as possible in what I have to say, that I may not occupy too much space in your valuable book, or too much of your readers' time ; but I am obliged to take some notice of this publication, in common justice to myself, and because I think I have long since proved that some of the author's practical observations are erroneous.

It will, I think, be sufficiently obvious to most of your readers, (if they will do me the favour to refer to the 15th volume of the Medical and Physical Journal, page 8,) that "the plan," which, he says, "he has now the honour to submit to the profession," has in reality nothing new in it; and that it has been almost entirely taken from my fracture-machine, or bed,—a drawing and description of which they will find at the place referred to. It is not a little curious, too, that the ground of argument in support of the method of treatment is almost literally the same as mine; and be it remembered that my communication to that Journal is dated 25th October, 1805.

A year and a half after this, (viz. 6th April, 1807,) a letter was published by Sir James Earle, giving an account of a fracture-bed invented by his son, and the history of a case where both thighs were fractured; a parallel case to the one which led to the invention of mine.

It is curious that even here the argument is so similar to mine, (see Sir James Earle's Letter, page 11,) that the first idea would be, that my Paper had been previously read by the writer. Under this impression, and observing that no notice was taken of my bed, ("Ad ogni uccello, suo nido è bello,") I wrote to Sir James Earle, complaining of the neglect; from whom I received the following letter. The original is now before me.

"Sir,—The construction of the bed, an account of which you say you have seen, entirely originated from the desire my son felt to give relief to the poor fellow, who was the subject of the case related in the same pamphlet. It was entirely his own invention; and I was much pleased with the ingenuity and utility of it. I added the bar to it, which I conceived would make it more extensively useful. I shall order my bookseller to send me the volume of the Medical and Physical Journal you mention, as I have not yet seen it. As your account which you mention of your machine has appeared so long since, I should be surprised that is not more in use; but that I know, and find, that what is not wanted in families every day is disregarded.

"It is surprising that the bed-ridden have been so long neglected.

"I have the honour to be, Sir, your most obedient servant,

(Signed) "JAMES EARLE."

"*Hanover-square; 9th January*, 1808."

"Since the above was written, I have seen both your accounts of your contrivance: it appears very ingenious, but, as you observe, much more complicated than my son's.

This letter places the fact beyond all reasonable doubt, that Mr. Earle must have seen, about that time, the account of my apparatus; and, after a lapse of fifteen years more, he publishes—what I think most unprejudiced and competent observers would consider *my* invention, as his own. He has, indeed, made some alterations.

I, too, have made great improvements, as I think, in mine, but *without altering the principle*, and which greatly simplify the application, and increase the power and accommodation of the instrument.

My first invention, however, not having been "rewarded by the Society of Arts, &c. with their large gold medal;" and not, so far as I know, having excited the attention of surgeons generally, till Sir Astley Cooper did me the favour lately, in his work on Dislocations and Fractures, to mention my case of fractured spine. My first invention not having produced much attention, "neither pride nor modesty" would permit me to make any more attempts to call it forth; or I should long since have had great pleasure in communicating my improvements to the medical world.

Having so far done justice to myself, or at least given the profession some opportunity of forming an opinion upon the subject, I now come to the investigation of some of his "Practical Observations." Mr. Earle observes, that "the principle of the double inclined plane is excellent, and, with certain modifications and additions, it will probably be found the most eligible mode of treating all fractures of the thigh."

Had he taken the trouble to look at page 121, vol. 26, of the Medical and Physical Journal, he would, perhaps, have entertained some doubt of the truth of this position: he would there have found a diagram explaining, as I think, pretty clearly, one description of fractured thigh, in which the use of the double inclined plane would most likely prove injurious, notwithstanding what is afterwards advanced in the same paper. The diagram shows that, when the fracture is in the shaft of the bone, within a certain distance of its lower extremity, if the fracture be not in a direction for the lower fractured portion to rest *upon* the upper,—i. e. if the fracture, for instance, be oblique, and upwards and backwards, when extension is made by pressure upon the calf of the leg, the action of the extensor muscles of the leg upon the patella *will occasion a depression of the lower fractured portion of the bone.*

The diagnosis, when the patient is placed upon the double inclined plane, is extremely obvious; the patella of the fractured limb is much less prominent than the other, and the depressed extremity of the bone may be *felt* in the ham, when any extension has been employed, between the *flexor* tendons of the leg. (See *Medical and Physical Journal*, vol. 26, page 121.)

Mr. Earle says, page 133, that he has not found it necessary to use any splints even in fractures of the shaft of the bone; but, if requisite, they may be added. The fact is, that Mr. Earle's alteration of my bed has made it no easy matter to apply splints to any purpose: he is therefore content, if possible, to do without them. Splints form an essential part of my fracture-boxes, are manageable with the greatest ease, and are of important service.

There is not unfrequently, in fractures above the middle of the bone, a tendency to incurvation at the knee, which lessens the proper coaptation of the broken parts of the bone. This is best managed by an additional small cushion of wool, between the inside of the knee and inner splint; and another above the fractured part, between the thigh and outside splint. Splints also support the limb through its whole extent, from the knee to the great trochanter; for, when the outside splint is applied to the other thigh, a strap across from one outside splint to the other, by acting upon the upper portion of both thighs, becomes a sort of pelvic bandage, affording the best means of perfect coaptation in cases of fracture of the cervix femoris; and, if boney union in fractures within the capsular ligament be in the power of nature, this will, in the best manner, assist her operations.

Whenever a splint upon the upper surface of the limb may be found necessary, the same straps and buckles which fix the lateral splints will fix this. The limb, thus inclosed, is kept so steadily to its proper position, as to diminish the pressure upon the calf of the leg, the part most likely to be uneasy.

With regard to complexity, (see "Practical Observations," page 103,) I do not see how a variety of positions, and the necessary accommodation which great differences of length and bulk require,—from seven years old to the largest adult size,—can be well ordered without a complex instrument, or that there can be any objection to it upon this account—*if it work well.* Few contrivances are more complicated than a watch; and, if one be required to keep good time, of course it must be more expensive than a less perfect and less useful machine.

*Cheshunt, Herts; 23d August*, 1824.

